# Polycaprolactone/α-cyclodextrin polyrotaxanes with cellular uptake enhancing properties

**DOI:** 10.1039/d4tb02451f

**Published:** 2025-01-31

**Authors:** Gergely Kali, Alexander H. Mayer, Dennis To, Martyna Truszkowska, Anna Seybold, Doris Elfriede Braun, Raphael Plangger, Markus Gallei, Andreas Bernkop-Schnürch

**Affiliations:** a Center for Chemistry and Biomedicine, Department of Pharmaceutical Technology, Institute of Pharmacy, University of Innsbruck, Innrain 80/82 6020 Innsbruck Austria gergely.kali@uibk.ac.at andreas.bernkop@uibk.ac.at; b Department of Zoology, University of Innsbruck, Technikerstraße 25 6020 Innsbruck Austria; c Center for Chemistry and Biomedicine, Department of Organic Chemistry, Institute of Chemistry, University of Innsbruck, Innrain 80/82 6020 Innsbruck Austria; d Polymer Chemistry, Saarland University, Campus C4 2 66123 Saarbrücken Germany; e Saarene, Saarland Center for Energy Materials and Sustainability, Campus C4 2 66123 Saarbrücken Germany

## Abstract

Biodegradable poly(ε-caprolactone) (PCL) was rotaxanated with α-cyclodextrin (α-CD) and an α-CD/2-hydroxypropyl-α-CD (HP-α-CD) mixture. Stoppering was achieved using 2-mercaptosuccinic acid (MSA) *via* disulfide linkage. The structures of these polymeric supramolecular entities were confirmed by ^1^H NMR, with 75–80 wt% threaded CD, while the molar mass of the polyrotaxanes was around 18 kDa, determined by gel permeation chromatography. The aqueous solubility was as low as 20.2 ± 1.2 g L^−1^ for the α-CD-based polyrotaxane but considerably increased to 74.7 ± 6.0 g L^−1^ by the introduction of threaded HP-α-CD into the polymeric axis. Dethreading of the polyrotaxanes was triggered by the removal of the stopper molecules *via* disulfide-exchange reactions using glutathione. Additionally, the polyester axis proved to be fully degradable by lipase. Cellular uptake of these polyrotaxanes was investigated by flow cytometry and confocal microscopy. The results showed an almost up to 50-fold higher cellular uptake of polyrotaxanes than free CD. These disulfide end-stoppered polyrotaxanes of biodegradable PCL represent a promising tool for intracellular delivery of CDs and offer novel treatment possibilities for lysosomal storage dysfunctions.

## Abstract 2

Biodegradable, disulfide stoppered poly(ε-caprolactone)polyrotaxanes threaded with α-CD and an α-CD/2-hydroxypropyl-α-CD mixture showed an almost 50-fold higher cellular uptake than free CDs and decomposition in the cytosol triggered by glutathione.

## Graphical abstract

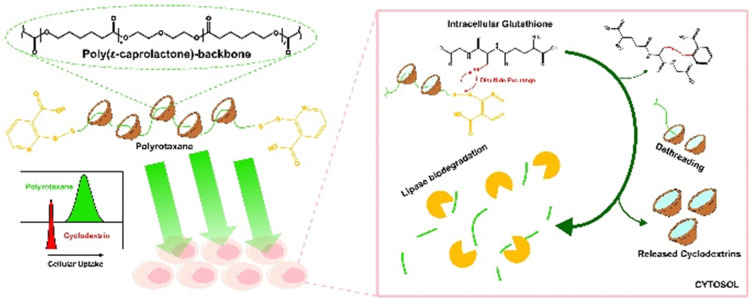

## Introduction

Supramolecular polymers, such as polyrotaxanes, have received huge interest in the last few decades.^1–5^ Polyrotaxanes consisting of a polymeric axis and threaded macrocycles, such as cyclodextrins (CDs), are stable supramolecular polymers in case the dissociation of the polymer and the macrocycle is prevented by bulky stopper groups along the chain or at the chain ends.^1,2^

Due to their unique interlocked structure, polyrotaxanes have several extraordinary properties and can be useful in biomedicine.^6–8^ The uptake of polyrotaxanes on several cell lines was highly enhanced compared to the parental CD, which is an important feature for drug delivery.^9,10^ Anticancer drugs, such as cisplatin,^9^ paclitaxel,^11,12^ and camptothecin^13^ as well as its derivatives,^10,14^ were conjugated to polyrotaxanes and successfully tested in cancer therapy. The decoration of polyrotaxanes with arginine moieties improves cellular uptake up to fivefold.^15^ Such cationic polyrotaxanes showed high efficacy in cellular internalization of condensed nucleic acids, even compared to branched poly(ethylene imine), the gold standard of gene delivery.^16–20^ The application of polyrotaxanes as therapeutics in lysosomal storage disorders (LSD) also showed promising results.^21,22^ The enhanced cellular uptake of these supramolecular polymers enables the delivery of a large number of CDs into the intracellular matrix, where dethreading supports the complexation of lipid substrates, normalizing their intracellular trafficking. One of the most investigated LSDs is Niemann–Pick disease, where cholesterol is enriched in the intracellular milieu, and polyrotaxanes of β-CDs are used for its treatment.^23^ GM1-gangliosidosis causes the enrichment of glycolipids in every organ, but mostly in brain tissue, and can be treated using α-CD, but its polyrotaxanes were never tested for this application.^24,25^

Despite these advantageous properties, polyrotaxanes are only used as luxury self-healing building and insulating materials due to their costly and time-consuming synthesis.^8,26–29^ Another drawback of polyrotaxanes for applications in the pharmaceutical industry is that they usually involve the poly(ethylene glycol) (PEG) polymeric axis for threading.^1^ The bioapplication of this polymer is highly undesired due to several problematic properties, such as anti-PEG IgM production or reactive oxygen species formation, described as PEG-dilemma.^30,31^ Recently, a straightforward method was described for the one-pot synthesis of polyrotaxanes *via rotaxa*-polymerization, which is limited to radically polymerizable hydrophobic monomers, such as isoprene, butadiene, or isobutylene, resulting in non-biodegradable products.^32–35^ As a result of the mentioned disadvantages, the widespread use of polyrotaxanes in medicine has not been reached yet, despite their extraordinary properties.^5^

A potential alternative to the above-mentioned polymeric axis is poly(ε-caprolactone) (PCL), a nontoxic and biocompatible polymer with a wide range of biomedical applications.^36–40^ This polyester's biodegradability also makes it attractive for drug delivery and tissue engineering.^36–38^ Until now, no polyrotaxanes of PCL have been described, but in some cases, pseudorotaxanes, without stopper moieties, were synthesized, and they tend to quickly dissociate.^39–43^

Therefore, the aim of this study is to synthesize end-stoppered polyrotaxane from PCL and α-CD or α-CD/HP-α-CD by introducing bulky 2-mercaptonicotinic acid end groups *via* the recently published one-pot two-step method.^44^ The enzymatic degradability of the polyrotaxane is investigated using lipase, and the effect of its interlocked structure on cellular uptake is studied by flow cytometry and confocal microscopy.

## Experimental

### Materials

Alpha-cyclodextrin (α-CD) and 2-hydroxypropyl-α-CD (HP-α-CD) were purchased from Cyclolab, Hungary. Poly(ε-caprolactone)-diol (PCL2000), cysteamine (≥98.0%), 1,1′-carbonyldiimidazol (CDI, ≥90%), 2-mercaptonicotinic acid (MNA, ≥98%), dimethyl sulfoxide-d_6_ (DMSO-d_6_, ≥99.9%), lipase (from porcine pancreas), fluorescein-isothiocyanate (FITC, ≥90%), dibutyltin dilaurate (DBTDL), 7-hydroxy-3*H*-phenoxazin-3-one 10-oxide sodium salt (dye content ≥ 75%), minimum essential Eagle medium (MEM), and Triton™ X-100 were ordered from Sigma-Aldrich, Austria. NucSpot Live 650 was obtained from Biotum, America. OptiMEM was received from ThermoFisher Scientific, Austria. All materials were used without further purification.

### Synthesis of thiol telechelic poly(ε-caprolactone)

The poly(ε-caprolactone) with reactive thiol chain ends was synthesized according to a previously published method, with some modifications.^45,46^ Briefly, 3.5 g of dihydroxy poly(ε-caprolactone) (PCL-diOH) (0.44 mmol) was dissolved in 95 mL of THF, and 2.77 g of CDI (17.1 mmol) was added to the solution. After the reaction was stirred for 24 h, the solution was concentrated using a rotary evaporator (40 °C, 370 mbar), precipitated twice into cold diethyl ether, and the product was dried under vacuum. Next, 3.00 g of PCL-carbamate intermediate (0.375 mmol) were dissolved in 45 mL of THF. After that, 0.58 g of cysteamine (Cys, 7.5 mmol) was added to this solution and stirred for 24 h. The reaction mixture was concentrated using a rotary evaporator (40 °C, 370 mbar), and the concentrate was precipitated twice into cold diethyl ether and dried under vacuum.

### Synthesis of poly(ε-caprolactone) α-cyclodextrin polyrotaxane (PCL-PRoX1)

The 2-mercaptonicotinic acid (MNA) stoppered PCL-based polyrotaxane with threaded α-CD was synthesized using a one-pot, two-step threading approach.^44^ First, 0.300 g of PCL dithiol (0.0375 mmol) was dissolved in demineralized water in a 100 mL round bottom flask. In parallel, 10 mL of a saturated α-CD solution was prepared. The pH of both solutions was set to 5 with 0.01 M HCl, and subsequently, they were mixed. The reaction system was stirred for 24 h at room temperature, and the intermediate polypseudorotaxane was obtained as a white precipitate. In a separate flask, the MNA dimer was prepared using 30% aq. H_2_O_2_ solution according to the literature.^47^ In the second step, the pH of the polypseudorotaxane suspension was increased to 7.5, and the MNA dimer was added. After 24 h of vigorous stirring, the aqueous polyrotaxane suspension was filtered. The solid was washed five times with demineralized water and subsequently freeze-dried for 2 days.

### Synthesis of poly(ε-caprolactone) α-cyclodextrin/2-hydroxypropyl-α-cyclodextrin polyrotaxane (PCL-PRoX2)

The PCL α-CD/HP-α-CD polyrotaxane was synthesized accordingly, using 0.725 g (0.746 mmol) of α-CD and 0.725 g (0.587 mmol) of HP-α-CD (weight composition 1 : 1). The resulting polyrotaxane was water-soluble, and therefore, the clear solution was dialyzed against demineralized water (Spectrum™ Spectra/Por™ 6 regenerated cellulose dialysis tubing MWCO 3.5 kD, from Fisher Scientific, Austria) for 3 days before freeze-drying.

### Characterization of the polymers and polyrotaxanes

The NMR measurements were performed on a “Mars” 400 MHz Avance 4 Neo spectrometer from Bruker Corporation (Billerica, MA, USA, 400 MHz) in DMSO-d_6_. For the measurements, around 5 mg of the dry samples were dissolved in 0.7 mL of deuterated solvent. The chemical shifts were reported in parts per million, and the center of the deuterated solvent, DMSO-d_6_, served as the internal standard (*δ* 2.49 ppm).

Fourier-transform infrared (FTIR) spectra of free CDs and polyrotaxanes were recorded using Bruker ALPHA FT-IR apparatus equipped with a platinum ATR (attenuated total reflection) module. The FTIR spectra were normalized to the intensity of C–O stretching vibrations at 1025 cm^−1^.

Gel permeation chromatography was performed using a Shimadzu Shim-Pack GPC-80MD column and an RID-20A refractive index detector at 25 °C in order to determine the molar masses and dispersities of the synthesized polyrotaxanes. The mobile phase was DMF supplemented with 1 g L^−1^ LiBr, with a flow rate of 1 mL min^−1^ using an LC-40D GPC pump. The calibration curve was based on linear PEG standards from Polymer Standards Service (PSS) of 0.238 to 31.7 kg mol^−1^.

The molar masses of the polyrotaxanes were determined by static light scattering (SLS) using a Zetasizer Nano ZSP (Malvern Instruments, Worcestershire, UK) with a laser wavelength of 633 nm at 25 °C. The polyrotaxanes were dissolved in DMF supplemented with 1 g L^−1^ LiBr with concentrations between 1000.00 and 31.25 mg L^−1^.

The MNA stopper content was determined by spectrophotometric measurement.^47^ Briefly, 0.5 mg of the two PCL-polyrotaxane samples were suspended in 500 μL of 0.5 M phosphate buffer pH 8, and reduced glutathione was added to reach a final concentration of 0.2% (m/v - mass of solute (g) in 100 mL of solution volume). After a 60-min incubation period, the absorbance of the supernatant was measured at 354 nm. The amount of MNA groups was calculated using a previously established calibration curve of MNA under the same conditions.

To determine the aqueous solubilities of the polyrotaxanes, 150 mg of the samples were dispersed in 0.6 mL of 0.1 M phosphate buffer with pH 7 and incubated at 37 °C in a thermomixer (Eppendorf ThermoMixer® C, Eppendorf AG, Hamburg, Germany) with a shaking speed of 1000 rpm. After 2 hours, the samples were centrifuged at 13 400 rpm for 5 min (MiniSpin®, Eppendorf AG, Hamburg, Germany), and 0.5 mL of the supernatant was withdrawn and lyophilized (Freeze Dryer Christ, Gamma 1–16 LSC, Germany), and the dissolved amount of polyrotaxane was quantified gravimetrically.

### Isothermal titration calorimetry (ITC)

All ITC experiments were performed with a MicroCalTM iTC200 System (GE Healthcare – Life Science) at 25 °C in 0.1 mM sodium phosphate buffer pH 8. The calibration, in order to determine complex formation, was carried out using aqueous native α-CD solutions at concentrations of 13 μM, 26 μM, and 130 μM in a sample cell (205.2 μL). After the first single injection of 0.5 μL, 19 injections of 2 μL 10 mM 1-hexanol were performed (0.5 μL s^−1^ injection rate, 150 s intervals between the injections, and 10 μcal s^−1^ reference power). The heat data were fitted with a one-site binding model to obtain comparative values for external calibration. The polyrotaxane samples were dissolved under the same conditions as described for free α-CD and titrated with 10 mM 1-hexanol.

### Powder X-ray diffraction (PXRD)

An X’Pert Pro diffractometer (PANalytical, Almelo, NL) was used for the study. This diffractometer, equipped with a PIXcel^1D^ detector, was operated with a Cu K_α1,2_ radiation source at 40 kV and 40 mA. The instrument was configured on a *θ*/*θ* coupled goniometer in a transmission geometry setup. Measurements covered a 2*θ* range from 2° to 40° with a step size of 0.013° and a duration of 400 seconds per step (multichannel mixed-signal).

### Biodegradation of the poly(ε-caprolactone) polyrotaxanes

The biodegradability of the PCL axes of the polyrotaxanes was examined using preactivated lipase in a digestive medium (5 mM CaCl_2_ and 150 mM NaCl containing 10 mM Tris buffer pH 7.0), as described previously.^48^ In brief, 2 g of lipase was dispersed in 20 mL of digestive medium and centrifuged at 13 500 rpm, at 4 °C for 20 min. For the biodegradation study, 3 mL of the supernatant of this lipase solution was mixed with 3 mL of polyrotaxane solution (20 mg mL^−1^) prepared in a digestive medium, and the pH was adjusted to 7.0. Subsequently, the mixture was incubated at 37 °C for 24 h. The subsequent degradation of PCL by lipase resulted in a drop in pH from pH 7.0, which was compensated by the addition of 0.01 M or 0.10 M NaOH solution at predetermined time points. Incubation was continued for up to 4 days and the degraded amount of ester was calculated from the amount of NaOH required for pH adjustment.

### Transmission electron microscopy (TEM)

The size and shape morphology of the two polyrotaxanes were evaluated by energy-filtered transmission electron microscopy (EFTEM).^49^ Saturated polyrotaxane solutions were prepared by dispersing 100 mg of the corresponding macromolecule in 1 mL of demineralized water, followed by incubation at 37 °C in a thermomixer (Eppendorf ThermoMixer® C, Eppendorf AG, Hamburg, Germany) with a shaking speed of 400 rpm and centrifugation at 13 400 rpm for 5 min (MiniSpin®, Eppendorf AG, Hamburg, Germany). The supernatant was transferred on 200 mesh Formvar/carbon-coated copper grids (Balzers Union, Liechtenstein). After drying, the samples were analyzed using a Zeiss Libra 120 (Carl Zeiss AG, Oberkochen, Germany). EFTEM images were taken using a 2 × 2 *k* high-speed camera (Troendle, Germany) and ImageSP software (Troendle, Germany).

### Cell viability studies

The cytotoxicity of the two PCL-based polyrotaxanes at concentrations of 0.15 and 0.30% (m/v) was determined using resazurin assay as previously described.^44,47,48^ Briefly, 20 000 Caco-2 cells per well were seeded in a 96-well plate in minimum essential medium (MEM) supplemented with penicillin/streptomycin solution (100 units/0.1 mg L^−1^) and 10% (v/v – volume of solute (mL) in 100 mL of solution volume) heat-inactivated fetal bovine serum (FBS). The cells were incubated for 4 days at 37 °C under an atmosphere of 5% CO_2_ and in a 95% relative humidity environment, and the medium was replaced on the second day. On the day of the experiment, the cells were washed with preheated 25 mM 4-(2-hydroxyethyl)piperazine-1-ethanesulfonic acid (HEPES) buffered saline at pH 7.4 (HBS) at 37 °C and the polyrotaxane test solutions in HBS, as well as pure HBS buffer as the positive control, and 1% (v/v) Triton™ X-100 in HBS buffer as the negative control, were added to the plate. The cell culture plates were further incubated at 37 °C in a 5% CO_2_ and 95% relative humidity environment for 4 and 24 h. After the incubation time, all the test solutions were removed, and the cells were washed with buffer. An aliquot of 0.1 mL of 2.2 mM resazurin solution was added to each well, followed by an incubation period of 2 h. The cell viability was calculated from the supernatant's fluorescence, measured at the excitation and emission wavelengths of 540 and 590 nm, respectively, using the following equation
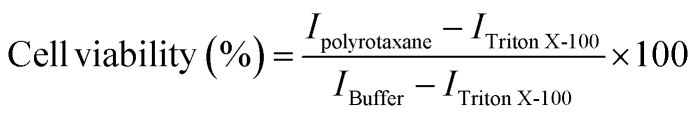
where *I*_polyrotaxane_, *I*_Triton X-100_, and *I*_Buffer_ stand for the fluorescence intensities of the samples, Triton™ X-100, and buffer, respectively. *I*_positive_ is given by the equation:Cell viability (%) = (*I*_sample_ − *I*_negative_)/(*I*_positive_ − *I*_negative_)

### Fluorescence labeling of the free and rotaxanated CDs

The CDs on the polyrotaxanes were labeled with FITC, as described previously.^44^ Briefly, 6.25 mg of FITC, as well as 375 μL of DBTDL, were added to 100 mL of 0.5% (m/v) DMSO solution of polyrotaxane. The mixture was stirred at room temperature for 24 hours in the dark. The fluorescently labeled polyrotaxanes were purified by dialysis against demineralized water under light protection using a Spectrum™ Spectra/Por™ 6 regenerated cellulose dialysis tubing (MWCO 3.5 kD) until no fluorescence was detectable in the dialysis medium. Native α-CD was modified in the same way.

### Flow cytometry

Cellular uptake studies on the Caco-2 cell line were carried out using the fluorescently labeled polyrotaxanes, while labeled native α-CD was used as control. The experiments were performed according to previously published protocols with minor modifications.^25,26^ Accordingly, the cells were seeded in a 24-well plate at a concentration of 2.5 × 10^4^ cells per well in a total volume of 500 μL MEM supplemented with 10% (v/v) heat-inactivated FBS and penicillin/streptomycin solution (100 units/0.1 mg L^−1^). The plate was incubated for 10 days at 37 °C in an atmosphere of 95% relative humidity and 5% CO_2_ to reach confluency and the medium was periodically replaced with fresh MEM. Fluorescently labeled α-CD and the polyrotaxanes were dissolved in an initial concentration of 0.01% (m/v) HBS. Subsequently, 100 μL of each sample was pipetted into a black 96-well plate, and the fluorescence intensity was measured with an excitation wavelength of 490 nm and an emission wavelength of 530 nm using a plate reader (Tecan Spark™, Tecan Trading AG, Switzerland). Samples showing higher fluorescence values were diluted with HBS afterward to ensure comparable fluorescence intensity for all samples.

On the day of the experiment, the cells were washed once with HBS before applying 500 μL of each sample to the cells and subsequently incubated at 37 °C. After 3 h of incubation, the samples were withdrawn, and the cells were washed once with HBS. Subsequently, detachment of cells was initiated by the addition of 150 μL of trypsin/EDTA 0.05%/0.02% and further incubation at 37 °C for 5 min. The reaction was terminated by the addition of 500 μL of MEM. Afterward, harvesting of the cells was performed by repeated, vigorous pipetting. The resulting cell suspensions were transferred to 15 mL Falcon tubes and centrifuged at 800 rpm for 4 min. The cells were subsequently washed once with 6 mL of cold phosphate buffered saline (PBS) and resuspended in a final volume of 500 μL of PBS. The cell suspension was eventually filtered through a cell strainer with a pore size of 70 μm for flow cytometry analysis. All samples were analyzed using an Attune NxT flow cytometer (Thermo Fisher Scientific, MA, USA). Fluorescence was excited by a blue laser (488 nm) and detected using the BL1 Channel (530 nm). Data analysis was performed using the software FlowJoTM v.10.10.0. The respective relative mean fluorescence intensity values (RMFI) were calculated according to the following equation:
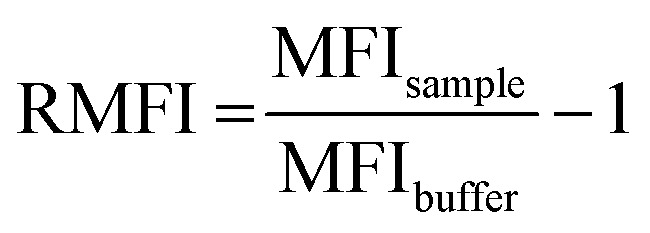


### Confocal laser scanning microscopy

Internalization of the samples by cells was further validated using a confocal laser scanning microscope (Leica TCS SP8) equipped with appropriate filter sets. Briefly, the cells were seeded in an eight-well chamber (μ-slide, Ibidi) at a density of 1 × 10^5^ cells per mL (3 × 10^4^ cells per well). Upon reaching confluency, the medium was replaced with OptiMEM for 30 minutes to minimize potential autofluorescence. Subsequently, the cells were exposed to a solution containing 0.1% (m/v) labeled native α-CD and polyrotaxanes dissolved in Opti-MEM for 3 hours. After exposure, the cells were washed three times with prewarmed Opti-MEM. Nuclei were stained using NucSpot Live 650 following the provider's recommendations (1 μL of NucSpot Live 650 1000× in DMSO diluted in 1 mL of OptiMEM) for 2 hours. The selected dye exhibited non-toxic properties, eliminating the need for a post-staining washing step. All fluorescence images were acquired under standardized conditions. Image processing was performed using ImageJ software, including the generation of *yz*- and *xz*-projections from 5 *xy* images of an image stack with a 0.2 μm *z*-step length. Spectral unmixing was applied to eliminate fluorescence bleed due to overlapping emission spectra between detection channels. Additionally, 2D image filtering was performed using a Gaussian filter.

### Statistical analysis

All experiments were performed at least three times. GraphPad Prism 5 (GraphPad Software, Inc., San Diego, CA, USA) was used to analyze the data. The one-way ANOVA and two-way Bonferroni multiple comparison tests were applied, and statistical significance was defined as *p* < 0.05.

## Results and discussion

### Synthesis and characterization

**Fig. 1:**
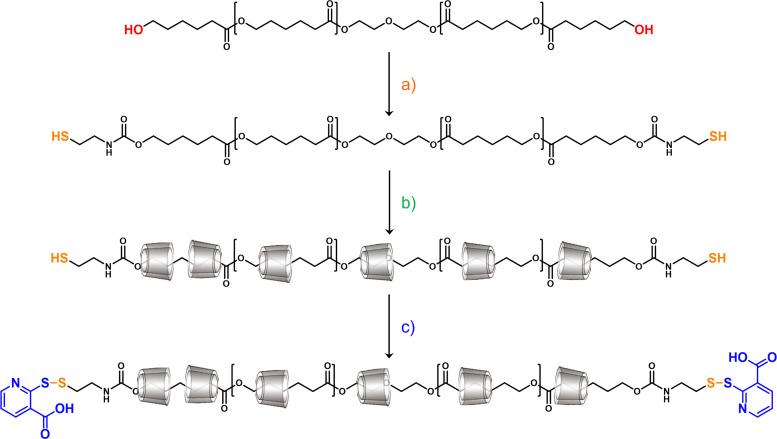
Illustration of the polyrotaxane synthesis: (a) CDI, cysteamine, THF, room temperature, 24 h; (b) α-CD or α-CD/HP-α-CD in demineralized water, pH 5, room temperature, 24 h; and (c) 2-mercaptonicotinic acid dimer, pH 7.5, room temperature, 24 h.

Poly(ε-caprolactone) (PCL) based polyrotaxanes were synthesized using the one-pot threading approach, as depicted in Fig. 1. Hydroxy telechelic PCL was thiolated at both chain ends using CDI and cysteamine, and α-CD and the α-CD/HP-α-CD 1 : 1 mixture were threaded on the polymer chain in water at pH 5. As the final step, the dimeric form of 2-mercaptonicotinic acid was added to the reaction solution in order to end-stopper the polyrotaxanes and hinder their decomposition.

**Fig. 2:**
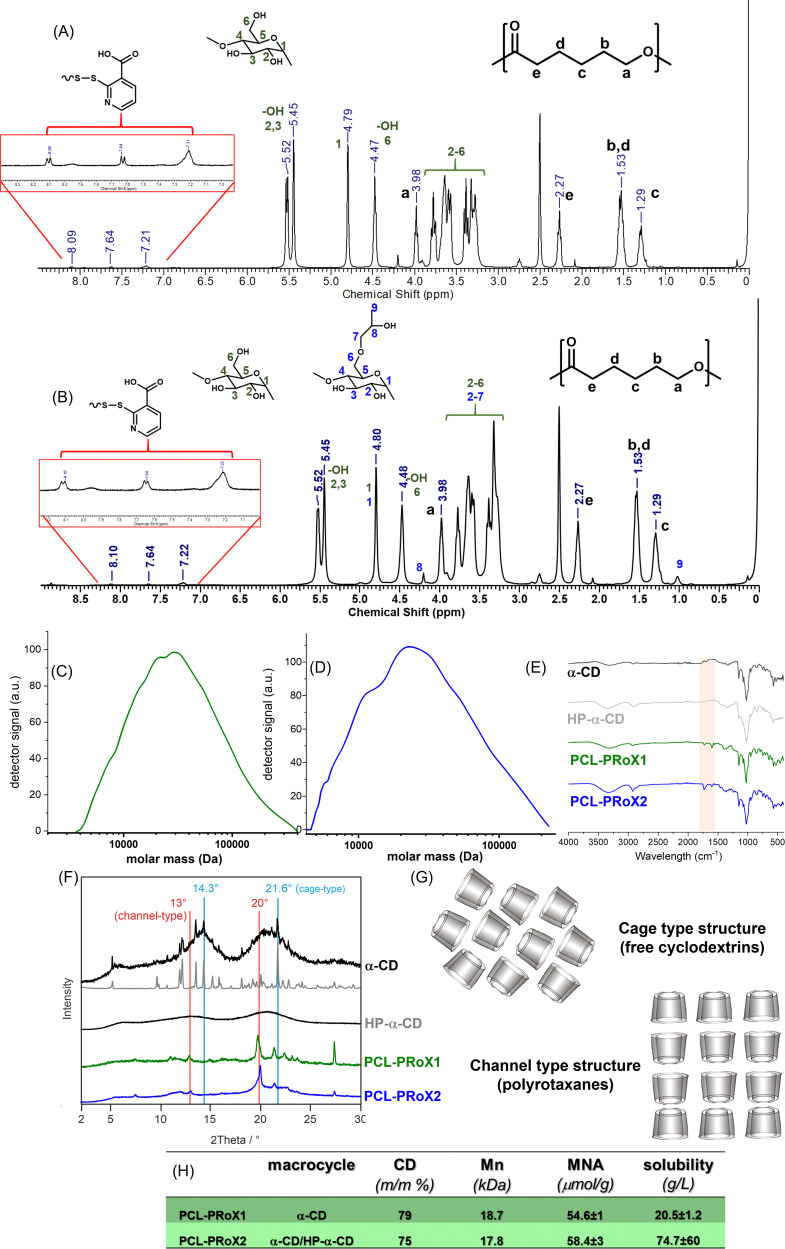
400 MHz ^1^H NMR spectra of (A) α-CD/PCL (PCL-PRoX1) and (B) α-CD/HP-α-CD/PCL (PCL-PRoX2) polyrotaxanes with the corresponding signals in DMSO-d_6_, molecular weight distribution of (C) α-CD/PCL (PCL-PRoX1) and (D) α-CD/HP-α-CD/PCL (PCL-PRoX2) polyrotaxanes, FTIR spectra (E) of α-CD (black line) HP-α-CD (gray line), PCL-PRoX1 (dark green line), and PCL-PRoX2 (dark blue line), (F) the powder X-ray diffraction patterns of α-CD (black), HP-α-CD (dark grey), PCL-PRoX1 (dark green), and PCL-PRoX2 (dark blue), while the crystalline hexahydrate of α-CD is shown in light grey (reference). The characteristic peak positions of the cage-type α-CD are indicated in blue, and those of the chain-type α-CD are indicated in red. Schematic representation (G) of the possible crystal structures of free and rotaxanated CDs. The main characteristics (H) of the synthesized poly(ε-caprolactone)/cyclodextrin polyrotaxanes are summarized.

In the ^1^H NMR spectra of both polyrotaxanes, the peaks of the methylene protons on the PCL axis at 1.29, 1.53, 2.27, and 3.98 ppm, together with the chemical shifts of the CDs at 5.55–5.45 ppm (OH-2/OH-3), ∼4.80 ppm (anomeric proton), and ∼4.45 ppm (OH-6), confirm the successful rotaxanation of the polymeric chain. The peaks in the aromatic region (≥7.00 ppm) belong to the 2-mercaptonicotinic acid (MNA) stopper molecules at the chain end, confirming the success of the stoppering reaction. The coverage of the polyrotaxanes was calculated using the integral values of the methylene protons of the PCL (a, b/d, c, or e, in Fig. 2A and B) and the anomeric protons of the CDs (1 and 1 in Fig. 2A and B). In both cases, the CD content of the polyrotaxanes was between 75 and 80% (m/v). The number average molar mass (*M̄*_n_) of the polymer axis and the polyrotaxanes could also be determined by endgroup analysis from the NMR spectra. The *M̄*_n_ of the PCL chain was determined to be between 4.1 and 4.3 kDa for both polyrotaxanes, which is a higher molar mass than that of parental PCL chains (∼2.0 kDa), most likely due to some disulfide bond formation between the PCLs with thiol end groups during the threading step. The *M̄*_n_ of the polyrotaxanes was calculated as 19.4 and 18.1 kDa for the macromolecules threaded with α-CD and an α-CD/HP-α-CD mixture, respectively. The molar mass of the two PCL-based polyrotaxanes was also determined by gel permeation chromatography (GPC). For PCL-PRoX1, 18.7 kDa (Fig. 2C), and PCL-PRoX2, 17.8 kDa (Fig. 2D), molar mass was measured, while the dispersities (*Đ*) were between 1.8 and 2.0. Finally, absolute weight average molar masses (*M̄*_w_) were determined by static light scattering (SLS). The results correspond well with the molar masses measured by NMR and GPC, as *M̄*_w_s of 17.4 and 16.7 kDa were determined for PCL-PRoX1 and PCL-PRoX2, respectively. FTIR measurements also confirmed the coexistence of the CDs and the polymeric axis, as shown by the CD spectra and the C=O stretch of PCL ester substructures at 1750–1580 cm^−1^ (Fig. 2E). The tiny peaks at around 1550 cm^−1^ in the FTIR spectra of both polyrotaxanes are attributed to the C=C stretching of the 2-mercaptonicotinic acid end-stoppers. Finally, the structure of the polyrotaxanes was also investigated by PXRD, and the powder X-ray diffraction patterns of the α-CDs, as well as the polyrotaxanes, are shown in Fig. 2F. For α-CD and HP-α-CD, a low crystalline hexahydrate^50^ and amorphous pattern, respectively, is detected. These patterns change after rotaxanation. In PCL-PRoX1 and PCL-PRoX2, the arrangement of the α-CD and HP-α-CD produces channel-like structures, as depicted in Fig. 2G, with characteristic reflections in the PXRD patterns (2*θ* = 13° and 20°). These reflections are markedly different from the crystalline and amorphous phases of the α-CDs. No α-CD hexahydrate diffraction peaks are detected (cage-type structure, 2*θ* = 14.3° and 21.6°), confirming the complete formation of the rotaxane as a result of the threading of CDs on the polymeric backbone.^51–54^

The free CD content of the products was also determined using isothermal titration calorimetry (ITC) in order to investigate the threading efficacy of the method. The free CD content of the polyrotaxanes was calculated by comparing the results with those obtained for α-CD solution. Almost no signal was detected in the thermograms of PCL-PRox 1 and 2; in both cases, the free CD content was below 5% (m/v), suggesting efficient threading and stoppering. The success of the stoppering reaction was also investigated by measuring the MNA content of the polyrotaxanes. In the case of PCL-PRoX1, the stopper content was 54.6 ± 14 μmol g^−1^, while for PCL-PRoX2, 58.4 ± 28 μmol g^−1^ MNA was detected. Furthermore, this measurement involves the MNA release by the interaction of the disulfide bond with glutathione (GSH); therefore, the responsiveness of the stopper molecules to this biomolecule, which is present in the intracellular milieu in high amounts, was demonstrated by this method. The removal of the stopper molecules resulted in CD release in the cytosol, as demonstrated for similar systems.^44,55–58^ This GSH sensitivity of the polyrotaxanes foreshadows their application as anticancer drug delivery systems due to the enriched GSH level in tumor cells compared to normal cells.^59,60^ These results clearly confirm the successful polyrotaxane formation from PCL and α-CD or α-CD/HP-α-CD by stoppering with the MNA dimer *via* GSH-responsive disulfide bond formation. The main characteristics of the synthesized polyrotaxanes of this work are summarized in Fig. 2H.

**Fig. 3:**
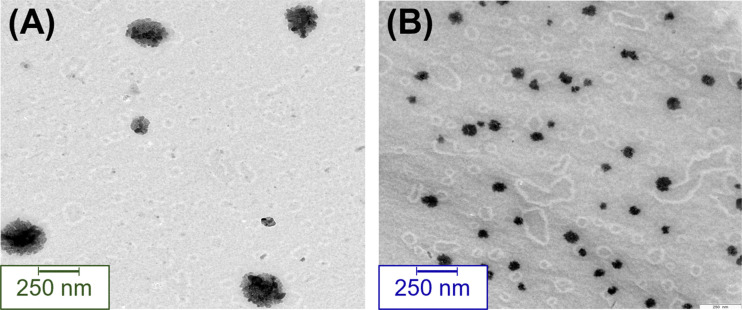
Energy-filtered transmission electron microscopy images of PCL-PRoX1 (A) and PCL-PRoX2 (B).

In several cases, polyrotaxanes also tend to self-assemble in an aqueous solution.^61–64^ The size, size distribution, and surface morphology of these self-assembled polyrotaxanes in demineralized water were determined by energy-filtered transmission electron microscopy (EFTEM). The results are depicted in Fig. 3. For both polyrotaxanes, spherical particles of different sizes were detected. In the case of PCL-PRoX1, the particle size showed multimodal distribution, as larger aggregates (255 ± 24 nm), as well as smaller non-uniform particles (92 ± 88 nm), coexist in the solution phase. The sizes are similar or, in the case of large aggregates, slightly higher than that of the previously described systems, mostly based on PEG.^64–66^ This deviation from the literature data is most likely due to the aggregation of the α-CD polyrotaxanes consisting of a more hydrophobic axis and higher threading efficacy. The latter results in a stiffer polymeric structure and enhanced hydrogen bonding between the oligosaccharides. However, PCL-PRoX2 polyrotaxanes, with some threaded HP-α-CD on the PEG backbone, self-assembled into uniform nanoparticles with a size of 85 ± 16 nm. The slightly smaller threading efficacy alone does not clarify this smaller size. The decreased hydrogen bonding, in the case of HP-α-CD compared to native α-CD, results in a 12-fold lower critical aggregation concentration for the hydroxypropylated derivative,^67^ which clearly influences the aggregation and the resulting particle size.

**Fig. 4:**
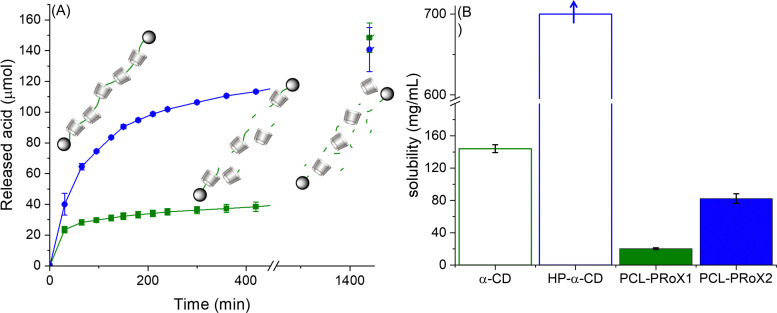
(A) Enzymatic degradation studies of α-CD/PCL (dark green square) and α-CD/HP-α-CD/PCL (dark blue circle) polyrotaxanes (10 mg mL^−1^) using preactivated lipase (6250 U mL^−1^) in a digestive medium (10 mM Tris buffer pH 7.0 with 5 mM CaCl_2_ and 150 mM NaCl). (B) Solubility of α-CD/PCL (dark green filled bars) and α-CD/HP-α-CD/PCL (dark blue filled bars) polyrotaxanes as well as α-CD (empty bars with dark green border) and HP-α-CD (empty bars with dark blue border) in 0.1 M PBS buffer pH 7 at 37 °C. Data are presented as mean ± SD *n* = 3 (***p < 0.001).

The great advantage of PCL over most of the other polymeric backbones for polyrotaxanes, such as PEG, is its biodegradability. Accordingly, biodegradation of the polymeric axes of the two polyrotaxanes by lipase is expected. The results of enzymatic degradation studies are depicted in Fig. 4. In the case of PCL-PRoX1, a rapid acid release was observed in the first 30 min, reaching almost 50 μmol h^−1^, but after that, it immediately slowed down to 10 μmol h^−1^ in the second 30 min and to 3 μmol h^−1^ in later stages. Similarly, PCL-PRoX2 followed the same degradation profile, but the enzyme hydrolysis was accelerated compared to PCL-PRoX1. After the initial 80 μmol h^−1^ burst acid release, the enzymatic hydrolysis of this polyrotaxane also slowed down, but to a lower extent to 50 μmol h^−1^ and later to 20 μmol h^−1^ in the next 30 min sections. After a total incubation of 420 min the degradation speed of PCL-PRoX2 was only around 3 μmol h^−1^. In the later stages, the rate of PCL-PRoX2 degradation decreases to 1.0 μmol h^−1^, most likely because of the end of enzymatic hydrolysis. Continuing the lipase degradation for another 4 days, the final amount of released acid is around 300 μmol, which is close to the amount of repeats of PCL in the measured polyrotaxanes (∼350 μmol). Therefore, it can be assumed that the polyester backbones are almost entirely hydrolyzed by lipase.

The difference between the degradation velocities is likely due to the fact that the second polyrotaxane contains HP-α-CD, which increases its water solubility.^44,68^ Therefore, the enzyme substrate, PCL, is readily accessible for lipase. Owing to the low solubility of PCL-PRoX1, its enzymatic degradation is limited mostly to the surface of the polyrotaxane particles, which determines the rate of the reaction in this case.

To confirm this hypothesis, the solubility of both polyrotaxanes was determined in phosphate buffer pH 7 at 37 °C. The resulting solubilities were compared to that of free CDs and are depicted in Fig. 4B. As was already expected and known from the literature, the native α-CD-based polyrotaxane has the lowest solubility of 20.3 ± 1.2 mg mL^−1^. Interestingly, this is a 2.3-fold higher solubility compared to the PEG-based polyrotaxane with threaded α-CD, synthesized by the same method described previously.^44^ This difference is most probably due to the slightly higher threading efficacy in the case of PCL and the different structures of the polymeric axes. In the case of the polyester, intramolecular hydrogen bonding between the carbonyl groups of the polymer backbone and the hydroxyl groups of the macrocycle is more favorable, while for PEG-based polyrotaxanes, the intermolecular hydrogen bonds lower the solubility. Threading of HP-α-CD on the backbone together with native α-CD highly enhanced the aqueous solubility of the polyrotaxane. The solubility of PCL-PRoX2 (74.7 ± 6.0 mg mL^−1^) is 3.7-fold higher than that of PCL-PRoX1. This is most likely due to the solubility difference between native and 2-hydroxypropylated α-CD, which was separately measured and depicted in Fig. 4B. It should be noted that the exact solubility of HP-α-CD could not be measured. Still the amount of this CD derivative was increased up to the concentration of 700 mg mL^−1^. Since the viscous solution was still clear, the solubility of HP-α-CD should be higher than this value.

### Cell viability studies

**Fig. 5:**
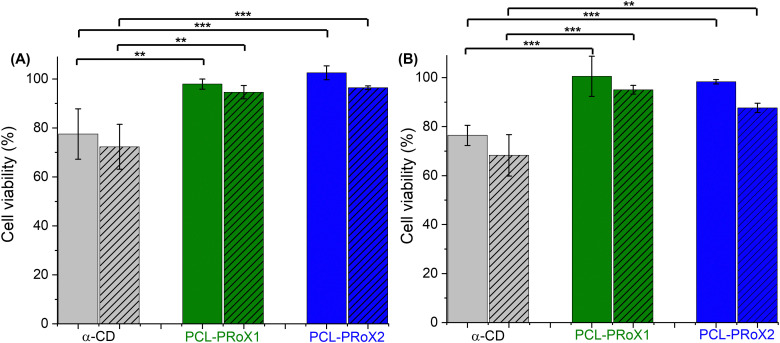
Cell viability of Caco-2 cells after (A) 4 h and (B) 24 h of incubation with α-CD (gray bars), α-CD/PCL (PCL-PRoX1, dark green bars), and α-CD/HP-α-CD/PCL (PCL-PRoX2, dark blue bars) at concentrations of 0.15% (m/v) and 0.3% (m/v, striped bars) at 37 °C. All results are shown as mean ± SD, *n* = 3 (***p < 0.001, **p < 0.01).

One of the most crucial parameters for excipients is their cytotoxic potential. Due to the biocompatibility of the two components, CD and PCL, no cytotoxicity is expected in the case of their polyrotaxanes. The cytotoxic potential of the two polyrotaxanes was investigated on Caco-2 cells at different concentrations and incubation times and compared with that of free CD. Results of cytotoxicity studies are depicted in Fig. 5(A) and (B) after 4 and 24 h of incubation, respectively. At the applied concentrations, 0.15 and 0.30% (m/v), no significant cytotoxicity was detected at either incubation time. In every case, the cell viability was higher than 80%, meaning these materials are generally considered as safe.^69^ Contrarily, the free α-CD shows slightly higher cytotoxic potential, reaching after 24 h incubation <70% cell viability for the concentration of 0.3% (m/v). These results confirm the advantage of using rotaxanated instead of free CD as an active pharmaceutical ingredient (API).

### Cellular uptake of polyrotaxanes

**Fig. 6:**
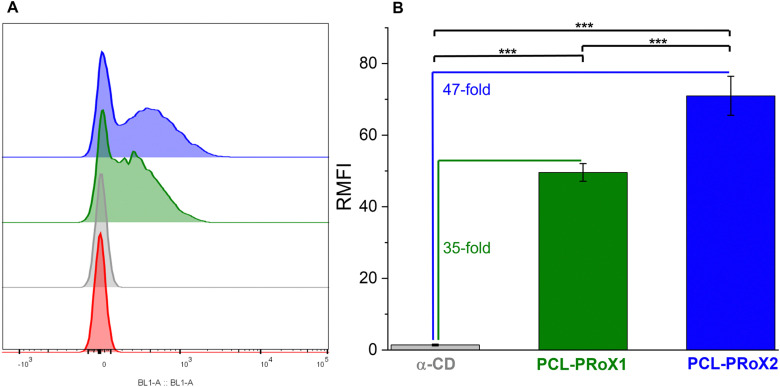
(A) Shift in fluorescence intensity (BL1 Channel): buffer only (red), α-CD (gray) α-CD/PCL polyrotaxane (PCL-PRoX1, dark green), and α-CD/HP-α-CD/PCL polyrotaxane (PCL-PRoX2, dark blue). (B) Analysis of cellular uptake of native α-CD (gray bar), α-CD/PCL (PCL-PRoX1, dark green bar), and α-CD/HP-α-CD/PCL (PCL-PRoX2, dark blue bar) polyrotaxanes at 37 °C. The relative mean fluorescence intensity (RMFI) values are presented for the Caco-2 cell line. The free and threaded CDs were labeled with fluorescein isothiocyanate. All results are shown as mean ± SD, *n* = 3 (***p < 0.001).

The application of CDs in the treatment of lysosomal storage diseases is not only determined by cell viability but also by cellular uptake, as these APIs have to enter the intracellular space to normalize intracellular trafficking. Therefore, cellular uptake of the polyrotaxanes was determined by flow cytometry and compared to that of free α-CD. For the comparison of the cellular uptake of the free CD and the polyrotaxanes, FITC-labeled oligo- and polymers were used. The similarity of fluorescence intensities was evaluated by measuring those of all the labeled products in the same concentration range in HBS buffer. The results of the flow cytometry analyses of the CD, PCL-PRoX1, and PCL-PRoX2 for Caco-2 cells are shown in Fig. 6A. The considerable shift in the fluorescence intensity to higher values in cases of the supramolecular polymers, compared to free CD, confirmed the improved cellular uptake for both polyrotaxanes. Quantification of the cellular uptake was carried out by calculating the relative mean fluorescence intensity (RMFI) values, depicted in Fig. 6B. In the case of PCL-PRoX1, a 35-fold higher cellular uptake was determined, compared to free α-CD. Cellular internalization was further enhanced, and 47-fold enhancement was achieved in the case of PCL-PRoX2 polyrotaxane over α-CD. These results show remarkable cellular uptake enhancement compared to free CDs and very similar to PEG-based polyrotaxanes, where 48- and 52-fold improvements were determined for α-CD/PEG and α-CD/HP-α-CD/PEG systems.^44^ This high and targeted cellular uptake of PCL-based biodegradable polyrotaxanes makes these materials attractive candidates for the treatment of lysosomal storage diseases.

**Fig. 7:**
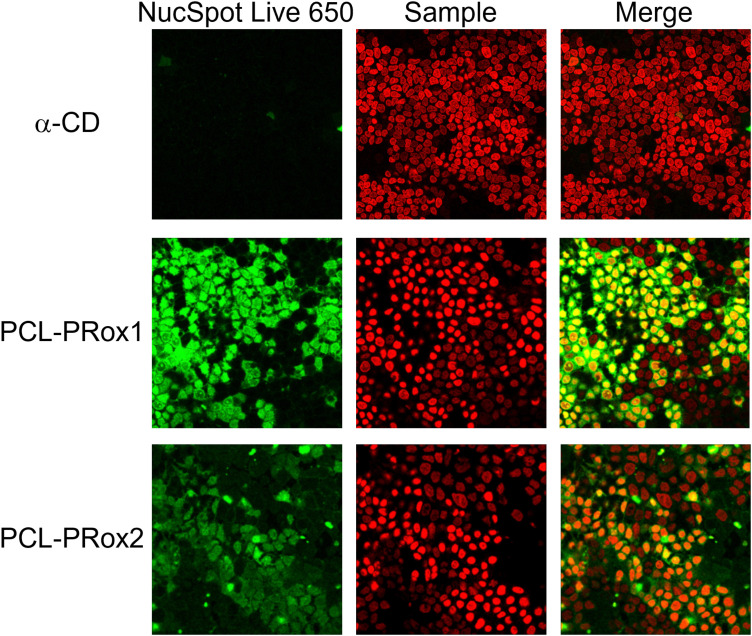
Visualization of cellular uptake of fluorescently labeled α-CD, α-CD/PCL polyrotaxane (PCL-PRoX1) and α-CD/HP-α-CD/PCL polyrotaxane (PCL-PRoX2) on Caco-2 cells using a confocal microscope. The nucleus was stained with NucSpot Live 650 (the scale bar on the figures is 20 μm).

In order to visualize the cellular uptake and confirm the results of flow cytometry measurements, confocal microscopy was carried out with FITC-labeled free CD and polyrotaxanes. Fig. 7 depicts the high-resolution images of the fluorescently labeled samples. For both polyrotaxanes, higher fluorescence was detected than in the case of free CD. In the case of PCL-PRoX1, the fluorescence marker is visible not only inside the cells but also around the nuclei. This is in correspondence with previous findings with native α-CD-based polyrotaxanes, which stick to the cell membrane in a high portion and are not properly internalized, likely due to the mechanism involving interaction with cell surface receptors or lipid components.^44^ The fluorescence signal of PCL-PRoX2 was detected inside the nuclei, confirming the success of internalization of this polyrotaxane by Caco-2 cells. The presence of the fluorescence marker within the cellular nuclei indicates successful traversal of the endosomal pathway by this polyrotaxane formulation. Consequently, the fluorescent dye is released into the cytoplasm, enabling its penetration into the nuclei. These observations underscore the divergent mechanisms of cellular uptake between α-CD/PEG polyrotaxane and α-CD/HP-α-CD/PEG polyrotaxane formulations. Specifically, PCL-RRoX1 predominantly interacts with the cell surface, whereas PCL-PRoX2 undergoes endocytosis, facilitating deeper penetration into the cellular interior.

The cellular uptake studies confirmed the potential application of biodegradable PCL-based polyrotaxanes in enhancing the cellular uptake of CDs as actives in the treatment of lysosomal storage disorders.

## Conclusion

Poly(ε-caprolactone) (PCL) based α-cyclodextrin (α-CD) and 2-hydroxypropyl-α-cyclodextrin (HP-α-CD) polyrotaxanes were synthesized for the first time, utilizing glutathione responsive disulfide stoppering. The structures of these products were confirmed by ^1^H NMR with CD content between 75 and 79% (m/v). The molar mass was, in both cases, around 18 kDa, determined by gel permeation chromatography. The degradation of the polyrotaxanes by lipase was highly dependent on their solubility, which together with their non-toxic properties makes them prominent candidates for pharmaceutical applications. Cellular uptake studies on Caco-2 cells exhibited an up to 50-fold enhancement in cellular internalization compared to free CDs. The determined biodegradability, low cell toxicity, and highly enhanced cellular uptake make these materials ideal for future applications in the treatment of lysosomal storage disease, GM1-gangliosidosis.

## Data availability

The data supporting this article have been included in the Experimental part.

## Conflicts of interest

There are no conflicts to declare.
